# Prediction of electrostatic properties of reservoir rock in low salinity water injection into carbonate reservoirs

**DOI:** 10.1038/s41598-023-36032-4

**Published:** 2023-05-31

**Authors:** Mohammad Parvazdavani, Mohammad Reza Khorsand Movaghar

**Affiliations:** Department of Petroleum Engineering, AmirKabir University of Technology, Tehran, Iran

**Keywords:** Chemical engineering, Geochemistry

## Abstract

Geochemical modeling along with chemical reactions is one of the challenges in modeling of low salinity water injection. The most important issue in the geochemical model is to determine the correct electrical charge distribution model and its tuning parameters. The composition of the rock as well as the candidate water used is effective in determining the type of model and its parameters, so that the tuning parameters are determined based on the history of zeta potential experiments. In this study, in order to determine the correct model of electrical charge distribution and its tuning parameters in carbonate rock samples, first, equilibrium samples of Candidate water with crushed rock are subjected to static zeta potential tests. Then, the diffuse electrical double layer model is used to determine the electrical charge of the rock/water and water/oil surfaces and to predict the zeta potential. In the following, by adjusting the tuning parameters of the model to match the prediction results of the model with the history of the laboratory data, the density of the carbonate rock surface, the equilibrium constants and the kinetics of the governing reactions are determined. The obtained results show that the range of error in zeta potential prediction by the model compared to the laboratory data is from 2 to 20%, which is within the acceptable range of the performance of electrical charge distribution models. Moreover, it could be observed that the error of prediction using DLM model is significantly less than the conventional models (CD-MUSIC and BSM) for different candidate water. Finally, the effect of calculated zeta potential changes is used to calculate the contact angle changes of low salinity water injection based on the coupling of DLVO theory and geochemical model. The results of the study prove that the prediction error is less than 5% compared to the results of the static wettability tests. Based on this, according to the good match between the model and the laboratory results, it is possible to determine the properties of surface sites in surface complexation models of carbonate samples using the proposed approach and the subsequent tuning data of the geochemical model.

## Introduction

The low salinity water injection has always been of interest in the enhanced oil recovery (EOR) process due to water availability and cheaper price than other materials. Seventeen mechanisms have been proposed so far for the low-salinity water injection, among them, change in wettability is mentioned as the dominant mechanism and leads to the change of wettability to less oil wet. This wettability condition caused lower residual oil saturation than the water wet and oil wet ones. Buckley et al. expressed wettability change as a result of interactions between oil and rock^1^. Berg et al. showed that it is possible to change wettability by injecting low salinity water^2^. Yousef et al. in 2011, together with Zekri et al. in 2012, stated that in carbonate rocks, by injecting low salinity water, the rock can become more water wet^3,4^. Adila et al. showed the effect of hybrid surfactant-LSWI/EWI on oil recovery from carbonate cores by coupling of surfactant flooding model with a geochemical model. They stated that Hybrid surfactant- Engineered Water Injection (EWI) combines the benefits of both surfactant and engineered water where the first controls the rate of oil production while the second controls the ultimate oil recovery achieved^5^.

The change in wettability is actually the result of the activation of the electric double layer expansion mechanism, in which low salinity water increases the thickness of water films and also makes it more stable. As a result of this phenomenon, the surface of the rock becomes more water wet and consequently, the oil production increases^6^. The stability of the water layer depends on the balance between the electrical double layer and van der Waals forces. Electrical double layer occurs when a charged surface is in contact with an aqueous phase. Ions with the opposite charge will have a higher concentration near the surface, while ions with the same charge will have a lower concentration. When the water–oil and rock–water surfaces have the same charge, they repel each other. The presence of water with a high salinity concentration conserves the charge distribution over the interface surfaces and leads to a reduction in the repulsive force. The amount of attractive or repulsive force between electrically charged surfaces (water/rock and oil/water) which is expressed based on the DLVO theory is directly related to the measured values of zeta potential. The amount of ionic strength as well as the type of ions present affects the amount of zeta potential and leads to a change in the thickness of the electrical double layer and as a result, changes in wettability. Many studies have expressed the effect of ion type, alkalinity (pH) and ionic strength on zeta potential and its effect on the attraction or repulsion of petroleum carboxylic groups to the rock surface^7–13^. Mahani et al. showed the trends for change of zeta potential versus pH in which in fixed of pH value, zeta potential decreased over the low salinity condition compared to FW for the limestone particles. Also, it was shown that the in case of SW, the change of zeta potential with pH is more pronounced compared to FW with a negative value of approximately −6 mV at pH value of 6.6^14^. Costa et al. found that the same behavior as well as limestone could be observed in case of sandstone sample. Solutions with high CaCl2 concentration (FW), the zeta potential becomes positive since alkaline earth ions are specifically adsorbed onto silica. They showed that in case of sandstone samples, zeta potential decreased over the low salinity condition compared to FW corresponding to fixed value of pH^15^. Alshakhs et al. investigated the wettability changes based on the contact angle calculation with the DLVO theory, which used laboratory zeta potential values for the disjoining pressure calculations and did not use a valid geochemical model to calculate the zeta potential^16^.

Two types of surface sorption models (SSM) and surface complexation model (SCM) have been used to consider the reactions of potential divalent ions (PDI) in the Stern Layer of the electrical double layer expansion mechanism to consider the charge distribution. One of the most important defects of SSM models is the failure to consider the charge distribution of rock surfaces sites, which is fixed in the SCM model. Surface complexation models generally operate based on a correct picture of the reactions governing water/rock or water/oil surfaces and are used to determine the electrostatic charge distribution and express the effect of water ionic compounds on absorption or repulsion of oil from the rock surface^17–23^. Sanaei et al. 2019 stated that zeta potential calculations based on the SCM can accurately predict the experimental data of oil/brine and brine/calcite zeta potential measurements^24^. Mosallanezhad and Kalantariasl (2021) used the compositional CMG model to investigate the critical rock and fluid interactions and dissolution/precipitation reactions were considered^25^.

Four common models of charge distribution in geochemical model are proposed in previous studies, one is the Constant Capacitance model (CCM) presented by Van Cappellen et al.^26^. In this model, they assumed that the surface reactions of the rock and the aqueous electrolyte solution are the same. Another model known as Charge-Distribution Multi-Site complexation (CD-MUSIC) was first proposed by Hiemstra and Riemsdi et al. This model has the ability to calculate zeta potential by introducing surface sites with different geometric coordinates (front, edge and side corners)^27^. Before presenting this model, Hiroth and his colleagues presented the diffused double layer model (DLM) to describe the electrokinetic behavior of calcite, which has provided a more accurate method for modeling the charge distribution of calcite surfaces and is more consistent with the experimental data of the electrostatic charge distribution of calcite surfaces^28^. The fourth model is the one presented by Heberling and his colleagues, which was based on equilibrium and non-equilibrium laboratory data of zeta potential^29^. Although all of these four models somehow express the charge distribution in electrical double layer geometry, the diffused double layer model is a better match with the physical reality of charge distribution and measured zeta potential data^30^.

Based on the correct performance of the electric double layer, the valid zeta potential values can be obtained for use in the DLVO theory. It is not possible to determine, calculate and compare the zeta potential of water/oil and water/rock surfaces separately in different ionic compounds without adjusting the laboratory parameters based on the charge distribution model. In this study, the aim is to calculate the zeta potential of the surfaces in different ionic compounds, which can be compared with the corresponding value in the laboratory and to validate the presented geochemical model. Based on the correct performance of the valid geochemical model in a certain range of rock type, injection water and operating conditions of pressure and temperature, it is possible to statically calculate the contact angle and check its changes in different injection water salinities^31^. Therefore, in this study, the main goal and novelty of this research was to complete the database of tuning parameters for the valid geochemical model (including the determination of the best charge distribution model), so that there is no need to repeat the experiments on carbonate (calcite) rocks under the specified conditions of the paper. Finally, the developed static geochemical model is verified by dynamic history matching of published core flooding experiments (Test No. 1 of Yousef et al.)^3^.

## Materials and methodology

### Materials

In the experimental tests, a limestone sample with low content of dolomite was used, the properties of core and its constituent compounds are shown in Table 1.

**Table 1:** Physical properties of the reservoir core containing the used crushed rock sample.

XRD Data	Permeability (mD)	Porosity (%)	Diameter (meter) in length (meter)	Core
99% Calcite, 0.8% Dolomite, 0.2% Chlorite	34.1	22.13	0.0864*0.0381	Limestone

The composition of candidate injection waters is also shown in Table 2. These waters have been used in single-phase coreflood experiments. NaCl, CaCl_2_.2H_2_O, MgCl_2_.6H_2_O and Na_2_SO_4_ mineral salts have been used to make synthetic candidate water. The calculation of required salts in 1 and 2 Liter of solution for SW/40 (4S) brine recipe is presented in table SI.2 of supplementary information. The calculation for other candidate injection brines is done and shown in Table 2.

**Table 2:** Ionic distribution of candidate injection waters in experiments.

Ionic concentrations (p.p.m)	Formation water (FB)	Seawater (SW)	diluted seawater enriched with sulfate (SW/40(4S))	sulfate-enriched seawater (SW(4S))
Na^+^	60,000	14,735	538	19,000
Ca^2+^	7100	500	13	510
Mg^2+^	1000	1640	41	1300
SO_4_^2−^	510	3579	1000	14,400
Sr	390	0	0	0
Cl^−^	108,000	24,108	603	23,000
TDS	177,000	44,562	2195	58,223

The selection of candidate water samples is done based on two main criteria which are approved by literature^32,33^. 40 times dilution have been selected based on the sensitivity analysis and optimization on change of contact angle versus salinity (mentioned in supplementary information, SI.1).

- Reduction of salinity by dilution (Forty-times dilution of seawater-SW/40)
- Increasing the potential divalent ion (PDI) that effects the wettability alteration (Four-times-sulfate seawater-SW(4S))

The properties of the oil sample used in the two-phase static contact angle tests are given in Table 3.

**Table 3:** Oil fluid sample properties.

Fluid properties	Amount
Saturate component (%)	39.17
Aromatic component (%)	48.30
Resins component (%)	7.04
Asphaltene component (%)	5.50
TAN (mg KOH/g oil)	0.25
P_b_ (Pascal)	12.44E + 6
GOR (Sm^3^/Sm^3^)	87.8
API at 15 $^\circ$C	30.0
Dead oil viscosity at room temperature (Pa.S)	14.59E-3
Dead oil density at room temperature (kg. /m^3^)	873.40

### Methodology

Based on the crushed samples of rock and candidate water compositions in Table 2, zeta potential tests have been performed to validate and adjust the tuning parameter of Double Layer Method (DLM) electrical charge distribution model provided by PHREEQC software in single-phase static conditions. In the following, to determine the wettability changes, contact angle tests have been conducted so that the process of wettability changes can be investigated based on different candidate waters in two-phase static conditions. Before presenting the results of the zeta potential tests, the standard method of measuring the zeta potential and then the contact angle have been described.

### Zeta potential measurement

**Figure 1:**
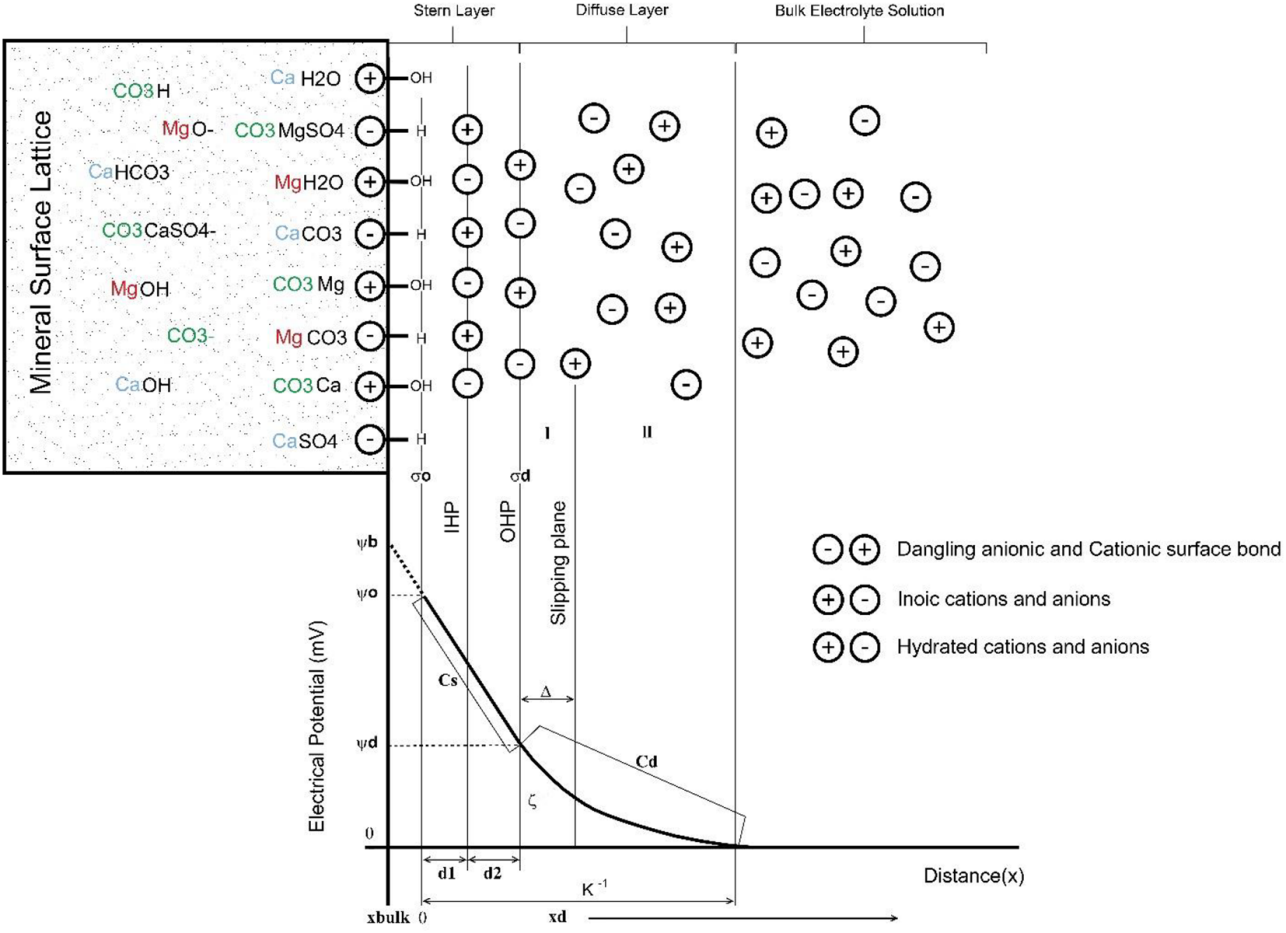
Schematic model of electrostatic charge distribution around the charged surface of the calcite rock containing low content dolomite and determination of zeta potential.

Figure 1 shows the drawing of electrostatic charge distribution model around the charged surface sites of the calcite rock containing low content of dolomite and definition of zeta potential. Zeta potential is very important to understand and control the properties of colloidal suspensions. Generally, the characteristics of a suspension can be identified by understanding how colloids interact with each other. Separation of charge at the interface between two phases is called electrical double layer. In this geometry, the attraction force from the negatively charged colloid causes positive ions to be absorbed and a layer is formed around the surface of the colloid. The layer with the opposite charge is called the Stern layer. Then, additional positive ions are absorbed by the colloid, but they are repelled by the Stern layer and other positive ions that try to approach the colloid. This dynamic balance leads to the formation of a diffused layer of oppositely charged ions. The high concentration of ions near the surface is gradually reduced by moving away from the surface of the colloid, and this process continues until the equilibrium with ions with the opposite charge in the solution (Bulk Electrolyte) is reached.

Similarly, due to the repulsive force of negative colloid, the concentration of negative ions decreases near the surface, and the concentration of these ions increases with increasing distance. The diffuse layer is visualized as a charged medium around the particle. The charge density at any point on the surface is equal to the difference between the concentration of positive and negative ions at that point.

Inside the diffusion layer, there is a boundary where the ions inside this boundary will move with the movement of the particle in the liquid, but the ions outside the boundary remain stationary. This boundary is called the slipping plane. On the other hand, the potential that exists between the surface of the particle and the liquid changes with the distance from the surface of the particle. This potential on the slip plane is called zeta potential. Two electrical layers are formed to neutralize the charge of the charged colloid. Instead, it creates an electrokinetic potential between the surface of the colloid and any point in the liquid mass. This voltage difference is a few millivolts and is known as surface potential (ψ potential). It depends on the surface charge and the thickness of the two electric layers.

**Figure 2:**
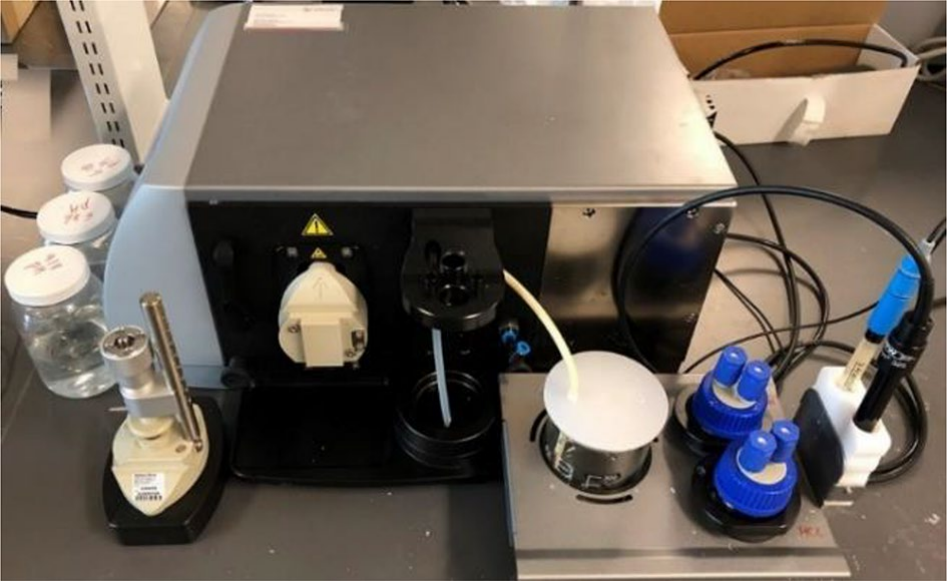
Schematic of the zeta potential measuring device and elements.

**Figure 3:**
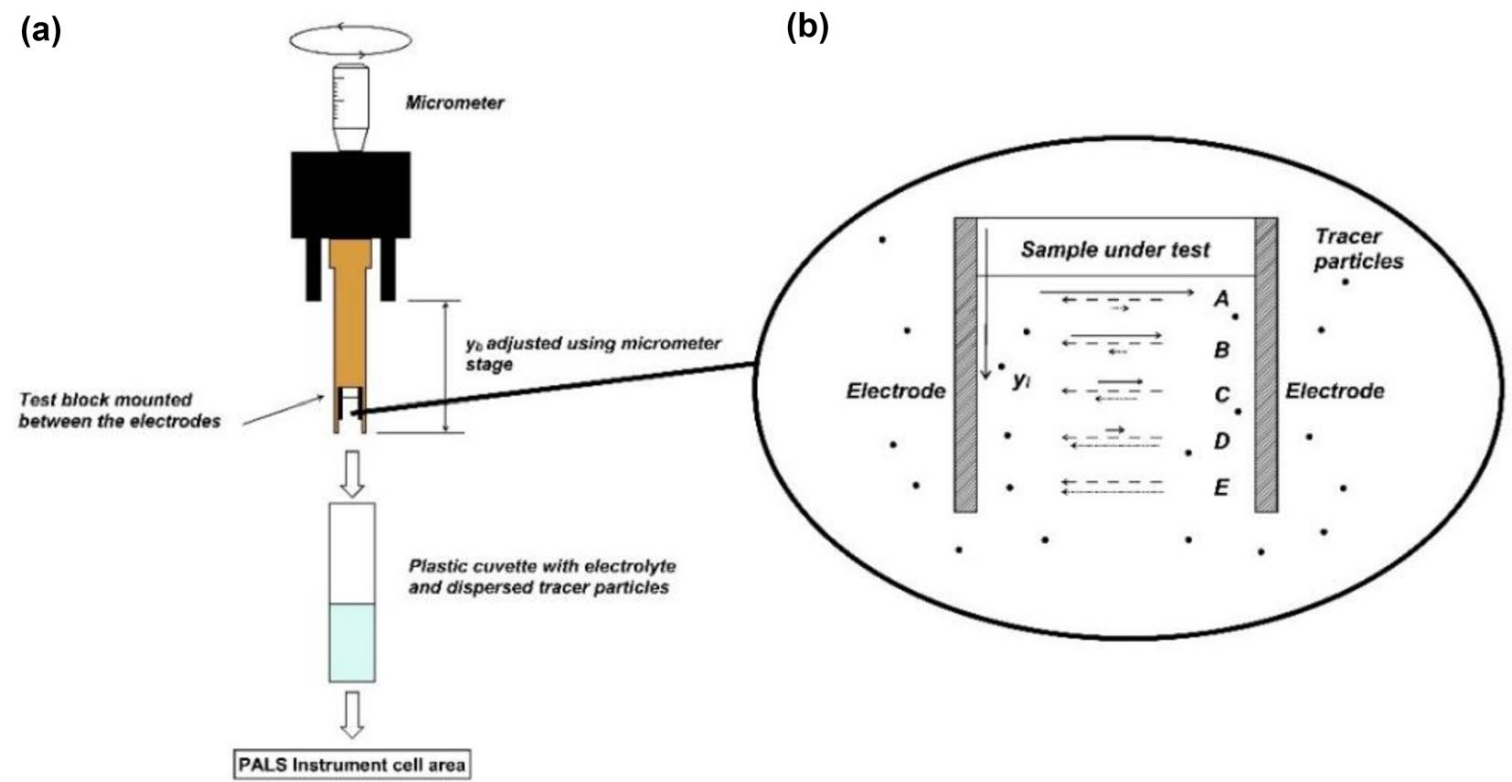
Procedure in determination of the electrophoretic mobility based on the dapper laser velocimetry method, (**a**) The sample surface mounting and the dip cell. (**b**) The measurement positions at displacement y_i_ from the test sample surface^34^.

The zeta potential of a particle can be obtained by Henry's equation (if the electrophoretic mobility of the sample ($${U}_{e})$$ is known) (Eq. 1).

1 $$U_{e} = \frac{{2\varepsilon \xi f\left( {k_{a} } \right)}}{3\eta }$$

where $\varepsilon$ is the dielectric constant, $\xi$ is the zeta potential, $f({k}_{a})$ is the Henry's function, which can be between 1 and 1.5 depending on the tested sample. $\eta$ is also viscosity. Therefore, according to the information of electrophoretic mobility, dielectric constant, viscosity and Henry's function, the value of zeta potential is obtained. For determination of electrophoretic mobility by device (Fig. 2), there are different methods. Three common methods are Dapper laser speed measurement, M3 and M3-PALS method. The first method is a common method in which the direction and amount of movement is a function of the particle charge, the suspension environment and the strength of the electric field. The velocity of the particles is determined by measuring the Doppler shift in the irradiated laser light. The speed of the particles is proportional to the electric potential of the particles in the shear plane or zeta potential. Therefore, the optical measurement of particle motion in an applied electric field is used to measure zeta potential (Fig. 3).

In this research, in order to perform zeta measurements, the samples were first set to an alkalinity level of 7.5 based on the buffer solution and then aged at this level for 7 days. This process was carried out at a temperature of 90 degrees Celsius and with a water volume of 100–250 cc as a dispersant. Also, before the start of the experiment, an ultrasonic device was used for 10 min with a power of 20 kHz and an output of 400 watts to homogenize the solution. Table 4 shows the necessary information for the preparation of the fluid sample to determine the zeta potential.

**Table 4:** Conditions on sample preparation for zeta potential measurements.

Parameter	Value
Alkalinity level of water (pH)	7.5
Aging time (days)	7
Temperature (^0^C)	90
Dispersant aqueous fluid volume (cc)	100 to 250
Time required for ultrasonication (Min)	10

### Contact angle measurement

In this research, the static contact angle method is used, in which a drop of a certain size is placed on the sample, then a high-precision camera captures the drop, the contact angle, and its three-phase line at the point of contact with the surface. Then, by image processing software, they are processed and angles are calculated in reservoir temperature of 90 $^\circ$C.

### Model description

In this section, the governing equations and relationships in geochemical modeling of electrical DLM model including the calculation of zeta potential are presented for carbonate surface sites. In the case of surface complexation reactions, kinetic-controlled reactions are considered based on existence of dolomite mineral^31^. Reaction rate ($${q}_{re})$$ is defined as following:

2 $${q}_{re} = {k}_{f}*{\left(1-\cap \right)}^{n},\cap = IAP/{K}_{sp}$$

where ${k}_{f}$ is the reaction rate coefficient and IAP is the product of ions activities. K_sp_ is the equilibrium constant of a chemical reaction and n is the power exponent of reaction rate. Equilibrium constant of other type of reactions is obtained by tuning the geochemical model and based on the database of PHREEQC (Version: 3.0.6-7757).

Equation 3 is presented to calculate the activity of surface sites.

3 $${\beta }_{i}=\frac{{N}_{i}}{\varphi CEC}$$

where ${N}_{i}$ is the molarity of ion i on the rock surface. Also, $\varphi$ and $CEC$ (Cation Exchange Capacity) represent porosity and ion exchange capacity of the carbonate surface including low content of Chlorite, respectively.

The surface potential is obtained based on the surface charge distribution during the Grahame equation obtained from the Gouy-Chapman theory, according to the following equation:

4 $${\sigma }_{0}=-{\sigma }_{d}=\sqrt{8000\varepsilon {\varepsilon }_{0}RTI}\mathrm{sinh}\left(\frac{{z}_{w}F{\psi }_{o}}{2RT}\right)$$

where σ_*o*_ and σ_d_ are the electrical charge density on the surface and the diffusion layer of the two electric layers in terms of charge surface unit (C/m^2^ (, respectively. $\varepsilon$ is the value of the dielectric constant of water and ${\varepsilon }_{0}$ is the permittivity constant in the vacuum space equal to (8.854 E^-12^ C^2^/J/m). Also, *z*_*w*_ is the charge of the background solution (usually equal to one). The global Faraday coefficient (F) is equal to 96,490 C/mol and temperature (T) is in degrees Celsius.

The amount of non-zero electrical potential in the stern layer of surface sites is obtained based on the total electrical charge of each surface site according to Eq. (5).

5 $${\sigma }_{0}=F\varphi \frac{CEC}{{A}_{\beta }{\rho }_{b}}\left(\sum_{i}^{No.\, of\, Surface\, Species}{\beta }_{i}{z}_{i}\right)$$

where ${\rho }_{b}$ is equal to mass density in kilograms per cubic meter. ${z}_{i}$ is equal to the valence or capacity of each ionic component. Based on this, according to relations (3), (4), (5), the value of ${\psi }_{o}$ on the surface of rock sample is obtained as the following relation:

6 $${\psi }_{o}=2RT/F*{sinh}^{-1}\left[\frac{F\varphi CEC}{{A}_{\beta }{\rho }_{b}\sqrt{8000\varepsilon {\varepsilon }_{0}RTI}}\left(\sum_{i}^{No.\, of\, Surface\, Species}{\beta }_{i}\right)\right]$$

The zeta potential (ζ) at the distance Δ from the outer Helmholtz plane (OHP) of the diffusion layer is obtained based on the surface charge distribution model of electrical double layer according to the following equation:

7 $${\upzeta =\psi }_{o}(\Delta )=2{k}_{B}T/e*ln\frac{1+\gamma {e}^{-k\Delta }}{1-\gamma {e}^{-k\Delta }}$$

Based on the exponential approximation of Eq. (6) and the Gouy-Chapman theory, the following equation is obtained for zeta calculation:

8 $${\upzeta =\psi }_{o}(\Delta )={\psi }_{o}{e}^{-k\Delta }$$

where k_B_ is equal to Boltzmann's constant and temperature is in degrees Kelvin. Based on the exponential relationship (8), the zeta potential changes from ψ*o* to ψ_*o*_/*e* at the level of Δ = κ^1^ (electric double layer wall). Therefore, the calculation of zeta potential in rock-water and water–oil interfaces (${\upzeta }_{r1}$ and ${\upzeta }_{r2}$ respectively) are obtained from relations (6) and (8), respectively.

**Figure 4:**
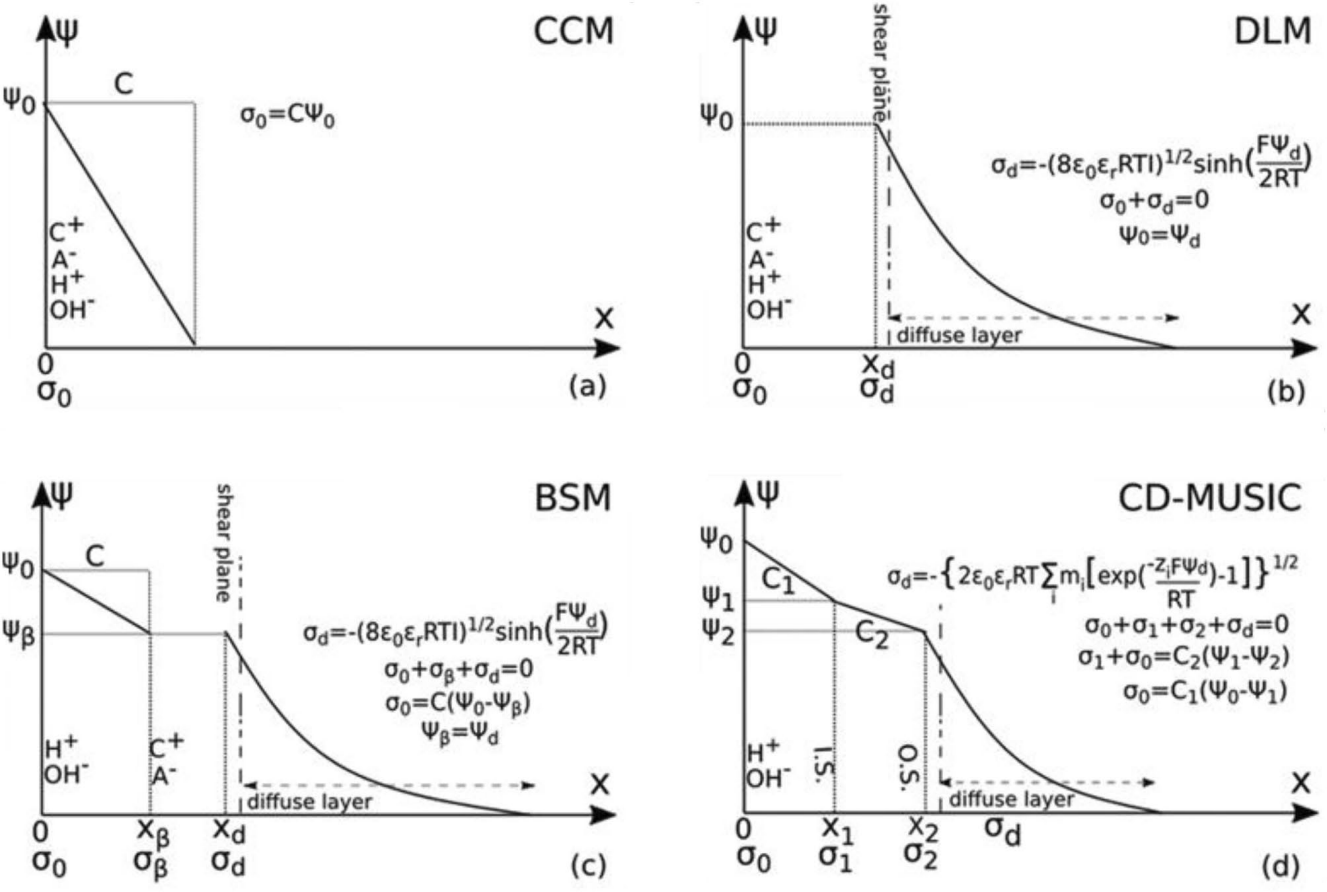
Comparison of different electrostatic charge distribution models of surface sites.

The comparative schematic of surface charge distribution in each of the commonly used models is shown in Fig. 4. As shown in the DLM model, the sum of the electrical charge density on the surface and the diffusion layer of the two electric layers is equal to zero, which is also shown in Eq. (4). Besides, the approximation of Eq. (7) has been used to estimate the zeta potential in the slipping plane. Excepting the CCM, other models consider the exponential decreasing trend for surface potential in diffuse layer.

By using the DLVO theory and calculating the zeta potential at the interfaces, the amount of contact angle changes can be observed based on salinity. First, it is necessary to state the computational steps of DLVO theory. In this theory, the changes in free energy between two rock/water and water/oil interfaces at a distance h from each other, which are filled with formation water, are used to evaluate wettability changes. This free energy is obtained from the following Eq. (9):

9 $$W(h)={\mathrm{W}}_{VDW}(h)+{\mathrm{W}}_{EDL}(h)+{\mathrm{W}}_{S}(h)$$

One of the main components in calculating the contact angle change is the calculation of the disjoining pressure of the two interfaces. Based on this, the disjoining pressure is obtained from the following relationship:

10 $${\prod }_{t}(\mathrm{h}) =\frac{\partial ({\mathrm{W}}_{VDW}\left(h\right)+{\mathrm{W}}_{EDL}\left(h\right)+{\mathrm{W}}_{S}\left(h\right))}{\partial h}={\prod }_{VDW}(\mathrm{h})+{\prod }_{EDL}(\mathrm{h})+{\prod }_{S}(\mathrm{h})$$

where VDW is related to van der Waals energy calculations, EDL is related to electric double layers and S is about structural energy. The positive and higher value of the disjoining pressure indicates the detachment of the oil phase from rock surface and the change of wettability to the water wet state. Also, due to the very low value of the structural energy at distances (h) less than 1 nm, this expression in relation (10) will be ignored. Using the Lifshitz theory, van der Waals energy is calculated based on Eq. (11):

11 $${\mathrm{W}}_{VDW} =AA/12\pi {h}^{2}, AA=3/4*{K}_{B}T(\frac{{\varepsilon }_{o}-{\varepsilon }_{w}}{{\varepsilon }_{o}-{\varepsilon }_{w}})(\frac{{\varepsilon }_{s}-{\varepsilon }_{w}}{{\varepsilon }_{s}-{\varepsilon }_{w}})$$

In which AA is Hamkar's constant in the range of 1E-21 to 1E-19. The relationship ${\varepsilon }_{o}$<${\varepsilon }_{s}<{\varepsilon }_{w}$ also exists between the dielectric coefficients of oil, rock and water. The potentials of the rock-water and water–oil interfaces have been used to calculate the energy term of the electric double layer in Eq. (10). The potential of the mentioned levels is generally obtained from the Poisson − Boltzmann equation (PBE) relationship as follows:

12 $$\frac{{\mathrm{d}}^{2}\uppsi }{\mathrm{d}{h}^{2}} =-e/{\varepsilon }_{w}\varepsilon *\sum {z}_{i}{\rho }_{i}exp(\frac{-{z}_{i}e\psi }{{K}_{B}T})$$

where e is the proton charge (1.6E−19 Coulomb). z_i_ and ρ_i_ are valence and charge density of ionic components, respectively. The Debye–Huckel assumption $\left(\frac{{z}_{i}e\psi }{{K}_{B}T}\right)<1)$ is used to solve the nonlinear PBE equation. Based on the analytical solution of the PBE equation and constant potential boundary conditions, Eq. (13) is expressed as the energy term of the electrical double layer of Eq. (10):

13 $${\prod }_{DL}(\mathrm{h})={n}_{b}{k}_{B}T\left[\frac{2{\upzeta }_{r1}{\upzeta }_{r2}\mathrm{cos}\left(kh\right)-{{\upzeta }_{r1}}^{2}-{{\upzeta }_{r2}}^{2}}{{(\mathrm{sin}\left(kh\right))}^{2}}\right]$$

where n_b_ is the density of water and k is the inverse of the Debye length, which can be calculated based on the ionic strength:

14 $${k}^{-1} =\sqrt{\frac{\varepsilon {\varepsilon }_{o}{K}_{B}T}{2{N}_{A}{e}^{2}I}} I=0.5\sum {{z}_{i}}^{2}{\rho }_{i}$$

where N_A_ is Avogadro's number (6.022E23 mol). I is the value of ionic strength based on mol/kg.

Based on the Young–Laplace relationship and the disjoining pressure integral from the lower range of the formation water film distance (h_0_) to the upper critical range of water thickness (h_p_), the contact angle is calculated according to the following relationship:

15 $$cos\theta =1+\frac{1}{\delta }{\int }_{{h}_{0}}^{{h}_{p}}{\prod }_{t}(h)dh$$

where $\delta$ is the water–oil surface tension in (mN/m).

All of the constants of Eqs. (1–15) are shown in Table 5 in summary.

**Table 5:** The constants of Eqs. (1–15).

Equation. No	Parameter	Value
1	$f\left({k}_{a}\right),$ Henry's function Constant	1–1.5
4	${\varepsilon }_{0},$ the permittivity constant in the vacuum space	8.854 E^-12^ C^2^/J/m
4	Faraday coefficient (F)	96,490 C/mol
11	AA, Hamkar's constant	1E-21 to 1E-19
12	e, the proton charge	1.6E-19 Coulomb
14	N_A,_ Avogadro's number	6.022E23 mol

## Results and discussion

**Figure 5:**
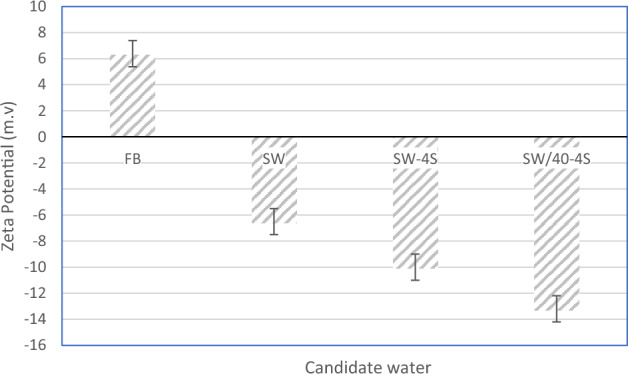
Zeta potential measured for Candidate injection water samples.

Based on the process of measuring zeta potential and preparing candidate samples, the zeta potential results for candidate water samples are presented in Fig. 5.

As shown in Fig. 5, with the increase of dilution and sulfate ion in the medium, the zeta potential decreases logarithmically and the slope of the curve is decreasing. The decreasing trend of zeta potential by application of low salinity condition is consistent with the corresponding trend of zeta potential regarding the salinity level in fixed value of pH (7.5 in Table 4). ^14^

In this part of the study, the aim is to develop a geochemical model and determine the parameters of the DLM model for the carbonate rock sample. It is possible to use the calculated values of zeta potential in DLVO theory relations based on the valid geochemical model. Therefore, the experimental data of zeta potential presented in Fig. 5 have been used to history match the geochemical model. In this regard, the tuning parameters in surface complexation of geochemical model are presented in Tables 6, 7, 8 for each of the surface sites. The reaction types are validated by Gorbani et al. in 2022^35^. Since, the equilibrium constant (Ksp) of calcite is very higher than dolomite (Table 7). Therefore, the precipitation/dissolution of dolomite is so slow and kinetically controlled reactions are considered only for dolomite (Table 8). It should be noted that the PHREEQC used formula (Eq. 16) is used to adapt the temperature change in calculation of equilibrium constant.

**Table 6:** Surface master species of carbonate surface sites used in PHREEQC.

Surface site	Master contributing component in the SCM	Density of stone sites (Site/ nm^2^)	contact surface (m^2^/g)	mass (g)
[Ca]	[Ca]OH	4.9	1.8233	2.31
[CO3]	[CO3]H	4.9	1.8233	2.31
[Mg]	[Mg]OH	35.45	0.3936	0.6816

**Table 7:** PHREEQC database used in geochemical reactions (equilibrium constants are at 25 °C).

		Aqueous reactions
1	${Ca}^{2+}+{SO}_{4}^{2-}=CaS{O}_{4}(aq)$	$\mathrm{log}k=2.25$
2	${Mg}^{2+}+{SO}_{4}^{2-}=MgS{O}_{4}(aq)$	$\mathrm{log}k=2.37$
3	${HCO}_{3}^{-}={H}^{+}+{CO}_{3}^{2-}$	$\mathrm{log}k=-10.39$
4	${HSO}_{4}^{-}={H}^{+}+{SO}_{4}^{2-}$	$\mathrm{log}k=41.07$
5	${CO}_{3}^{2-}+2{H}^{+}={CO}_{2}+{H}_{2}O$	$\mathrm{log}k=16.67$
		Dissolution/precipitation reactions
6	${CaC}_{3}\left(s\right)={Ca}^{2+}+{CO}_{3}^{2-}$	$\mathrm{log}k=-8.48$
7	$CaMg{\left(C{O}_{3}\right)}_{2}\left(s\right)={Ca}^{2+}+{Mg}^{2+}+2{CO}_{3}^{2-}$	$\mathrm{log}k=-17.09$
8	$CaS{O}_{4}\left(s\right)={Ca}^{2+}+{SO}_{4}^{2-}$	$\mathrm{log}k=-4.58$
		Surface complexation reactions (Green: [Ca]OH, Red: [CO3]OH, Purple: [Mg]OH)
9	$>Ca{H}_{2}{O}^{+}=>CaOH+{H}^{+}$	$\mathrm{log}k=-12.8$
10	$>Ca{H}_{2}{O}^{+}+{HCO}_{3}^{-}=>CaC{O}_{3}^{-}+{H}^{+}+{H}_{2}O$	$\mathrm{log}k=-5.65$
11	$>Ca{H}_{2}{O}^{+}+{HCO}_{3}^{-}=>CaHC{O}_{3}^{-}+{H}_{2}O$	$\mathrm{log}k=1.68$
12	$>Ca{H}_{2}{O}^{+}+{SO}_{4}^{2-}=>CaS{O}_{4}^{-}+{H}_{2}O$	$\mathrm{log}k=3.3$
13	$>Ca{H}_{2}{O}^{+}+{A}^{-}=>Ca{H}_{2}OA$	$\mathrm{log}k=0.4$
14	${>CO}_{3}^{-}+{H}^{+}=>C{O}_{3}H$	$\mathrm{log}k=5.48$
15	${>CO}_{3}^{-}+{Ca}^{2+}=>C{O}_{3}{Ca}^{+}$	$\mathrm{log}k=1.74$
16	${>CO}_{3}^{-}+{Mg}^{2+}=>C{O}_{3}{Mg}^{+}$	$\mathrm{log}k=1.74$
17	$>C{O}_{3}{Ca}^{+}+{SO}_{4}^{2-}=>C{O}_{3}Ca{SO}_{4}^{-}$	$\mathrm{log}k=3.3$
18	$>{{\mathrm{CO}}_{3}\mathrm{Mg}}^{+}+\mathrm{ QUOTE }{\mathrm{SO}}_{4}^{2-}=>{\mathrm{CO}}_{3}{\mathrm{MgSO}}_{4}^{-}$	$\mathrm{log}k=3.3$
19	$>C{O}_{3}H+{Ca}^{2+} = >C{O}_{3}{Ca}^{+}+{H}^{+}$	$\mathrm{log}k=-2$
20	$>{{\mathrm{CO}}_{3}\mathrm{Mg}}^{+}+{A}^{-}=>{\mathrm{CO}}_{3}\mathrm{MgA}$	$\mathrm{log}k=0.4$
21	$>{{\mathrm{CO}}_{3}\mathrm{Ca}}^{+}+{A}^{-}=>{\mathrm{CO}}_{3}\mathrm{CaA}$	$\mathrm{log}k=0.4$
22	$>C{O}_{3}H+{Mg}^{2+} = >C{O}_{3}{Mg}^{+}+{H}^{+}$	$\mathrm{log}k=-1.71$
23	$>MgOH= >Mg{O}^{-}+{H}^{+}$	$\mathrm{log}k=-12$
24	$>MgOH+{H}^{+}= >Mg{OH}_{2}^{+}$	$\mathrm{log}k=10.6$
25	$>MgOH{+CO}_{3}^{2-}+{H}^{+}=>MgC{O}_{3}^{-}+{H}_{2}O$	$\mathrm{log}k=15.4$
26	$>MgOH{+CO}_{3}^{2-}+2{H}^{+}=>MgHC{O}_{3}+{H}_{2}O$	$\mathrm{log}k=23.5$
27	${Na}^{+}+\frac{1}{2}>Mg{X}_{2}\iff \frac{1}{2} {Mg}^{2+}+>NaX$	$\mathit{log}k=-1.125$

**Table 8:** Reaction rate coefficients and exponents of the kinetic-controlled model in a limestone rock sample with low content of dolomite (Eq. 2).

Calcite Rock Type	k_f_ (g/cm^2^/day)	n
Aldrich calcite-70–100 (1E-6 m)	(3.7 ± 0.4) E-4	3.5 ± 0.2
Homegrown calcite-300–500 (1E-6 m)	(2.4 ± 0.9) E-4	2.9 ± 0.5
Homegrown calcite-500–700 (1E-6 m)	(1 ± 0.3) E-3	4 ± 0.6

16 $$\mathrm{log}\left(k\right)={A}_{1}+{A}_{2}T+{A}_{3}\left(\frac{1}{T}\right)+{A}_{4}\mathrm{log}\left(T\right)+\frac{{A}_{5}}{{T}^{2}}+{A}_{6}{T}^{2}$$

**Figure 6:**
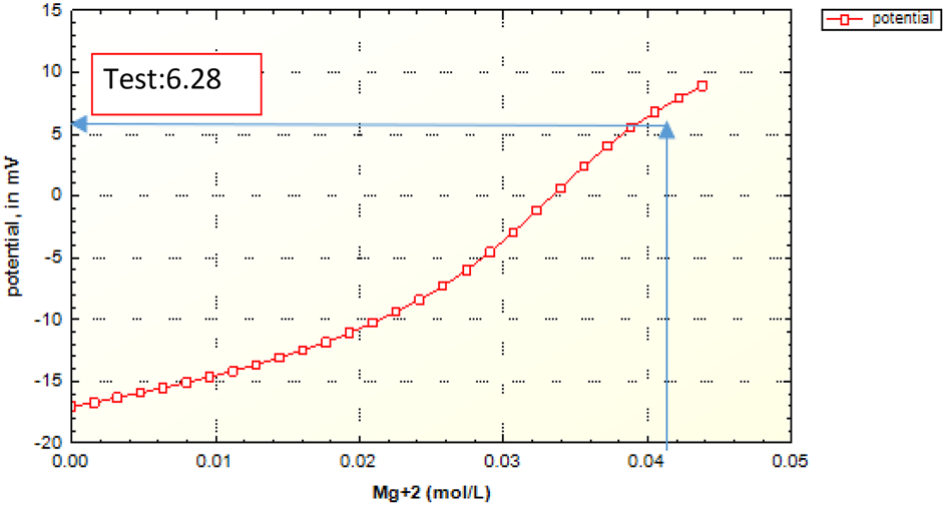
Zeta potential modeling results based on the magnesium concentration in the case of formation water (FB).

As shown in Fig. 6, the prediction value of zeta potential in case of formation water by the geochemical model is in good agreement with the laboratory data.

**Figure 7:**
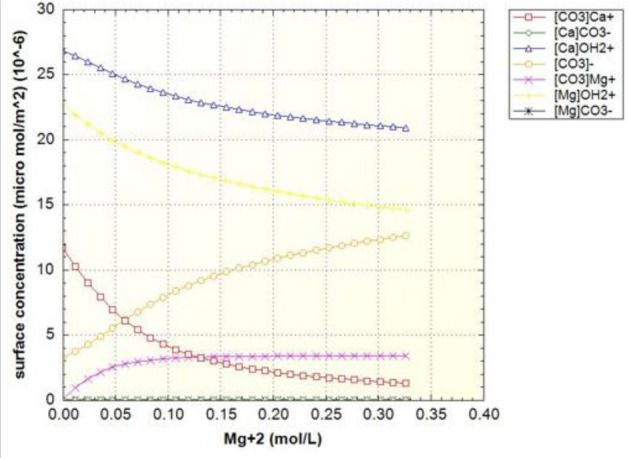
Surface sites concentrations of surface species in terms of magnesium concentration in the case of formation water (FB).

Figure 7 shows the density of surface sites, which are classified in three groups based on the main component of the surface site (Table 6). Due to the high composition of calcite in the crushed rock sample, the changes in the concentration of the carbonate site ([CO3] Ca) marked in red curve have been investigated. The concentration of this surface site should decrease due to the dissolution process, while the other carbonate site ([CO3] Mg) does not decrease due to the low rate of dissolution, but eventually the slope of its changes becomes zero. Based on the same performance of carbonate sites, the amount of concentration ([CO3]) in the environment is increasing and the slope of its increase decreases over time.

**Figure 8:**
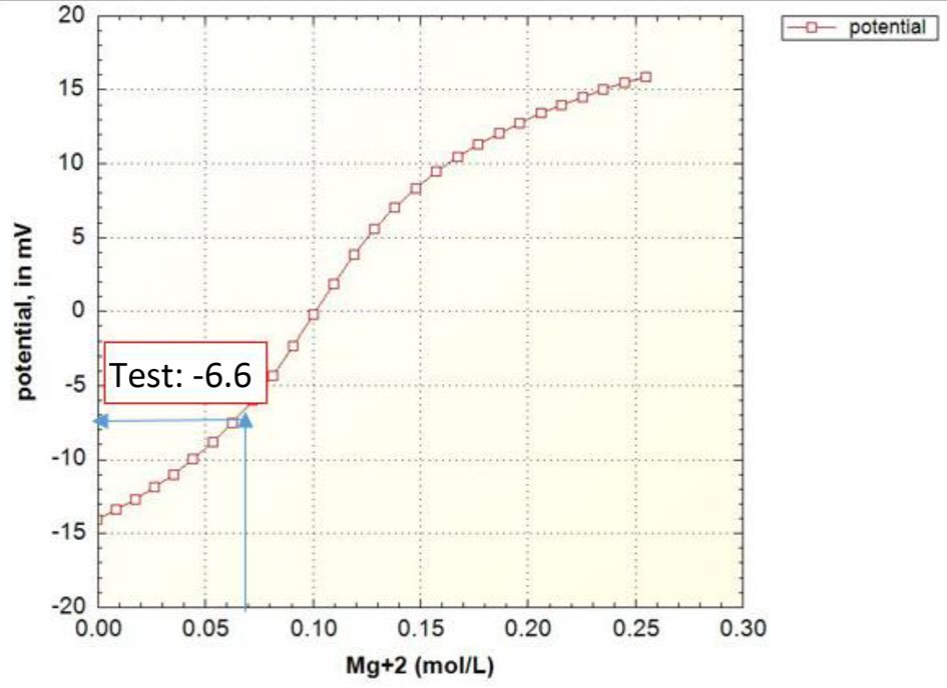
Zeta potential modeling results based on the magnesium concentration in the case of seawater (SW).

**Figure 9:**
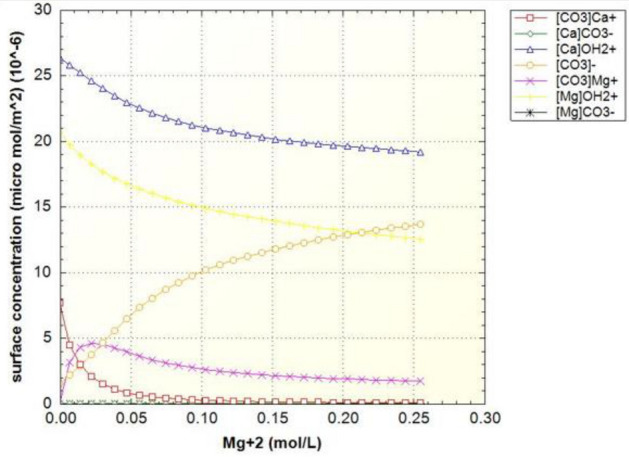
Surface sites concentrations of surface species in terms of magnesium concentration in the case of seawater (SW).

**Figure 10:**
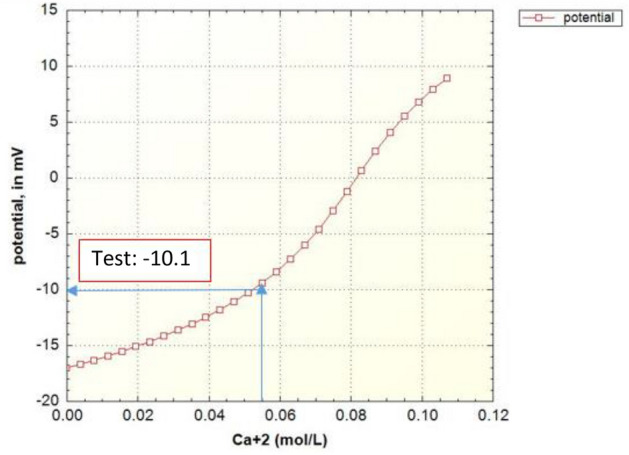
Zeta potential modeling results based on the magnesium concentration in the case of sulfate-enriched seawater (SW-4S).

**Figure 11:**
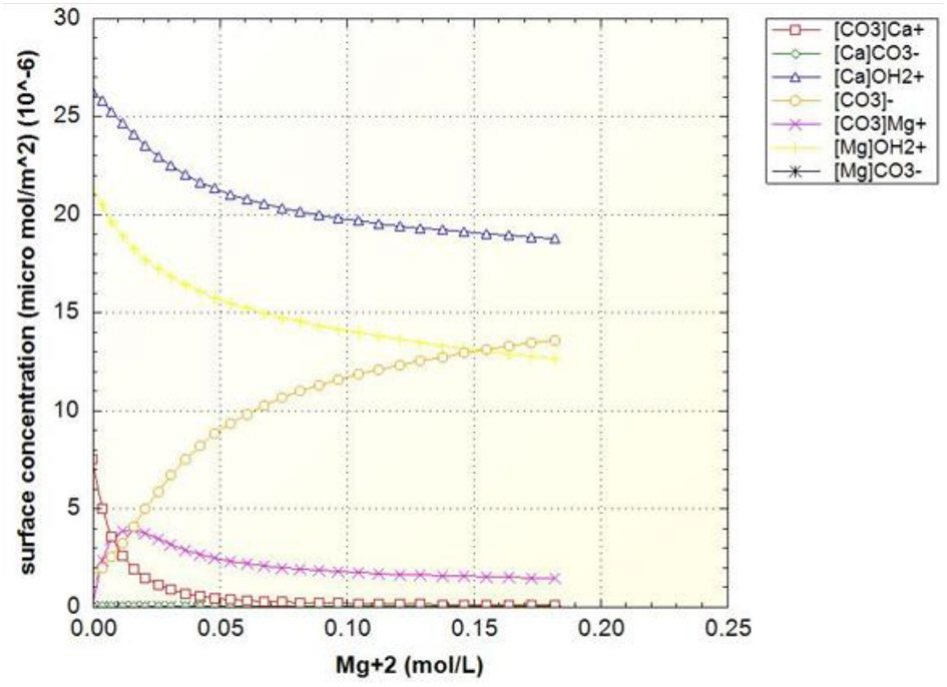
Surface sites concentrations of surface species in terms of magnesium concentration in the case of sulfate-enriched seawater (SW-4S).

**Figure 12:**
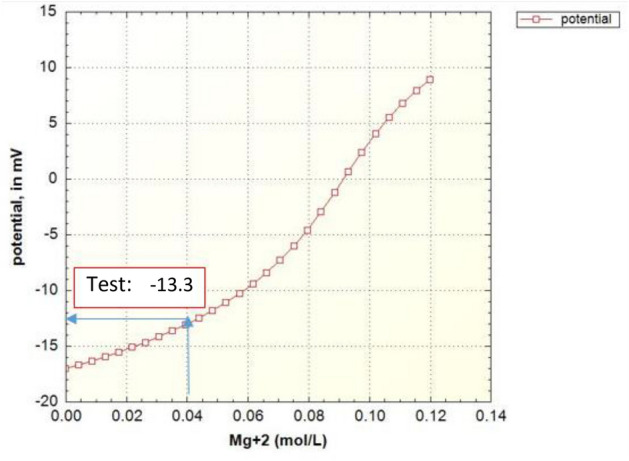
Zeta potential modeling results based on the magnesium concentration in the case of seawater diluted forty times and enriched with sulfate (SW/40-4S).

**Figure 13:**
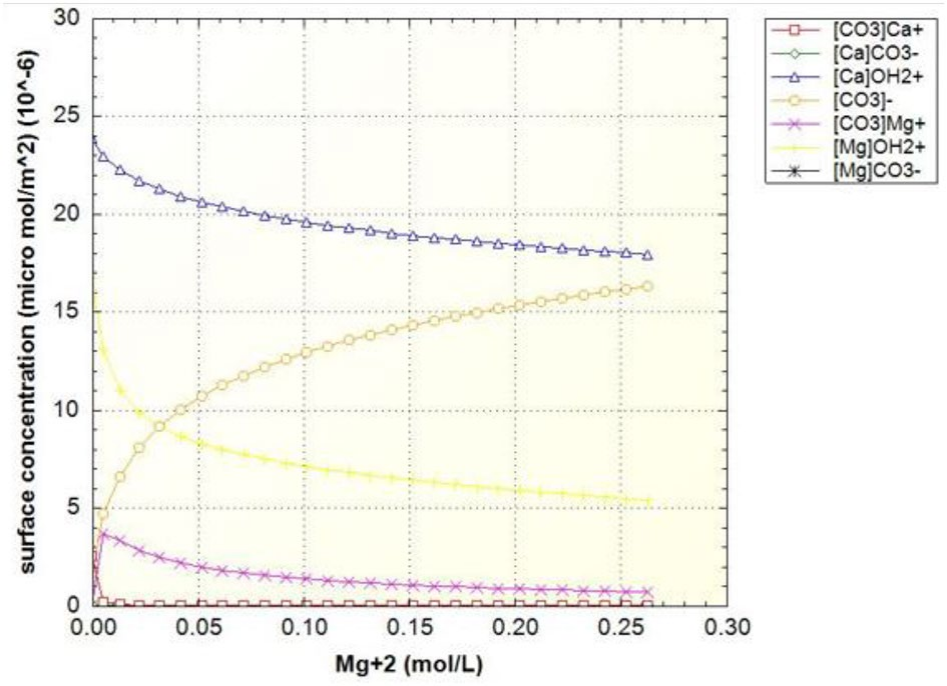
Surface sites concentrations of surface species in terms of magnesium concentration in the case of seawater diluted forty times and enriched with sulfate (SW/40-4S).

Figures 8, 9, 10, 11, 12, 13 show the corresponding prediction results of zeta potential along with the concentration of surface sites for the other candidate waters in Table 2.

As can be seen in Fig. 9, like the curve in Fig. 7, the amount of carbonate rock site should decrease, but this process occurs at a faster rate. The equivalent curves of zeta potential for samples of water enriched four times by sulfate and diluted forty times are also drawn in Fig. 10 to Fig. 13.

**Figure 14:**
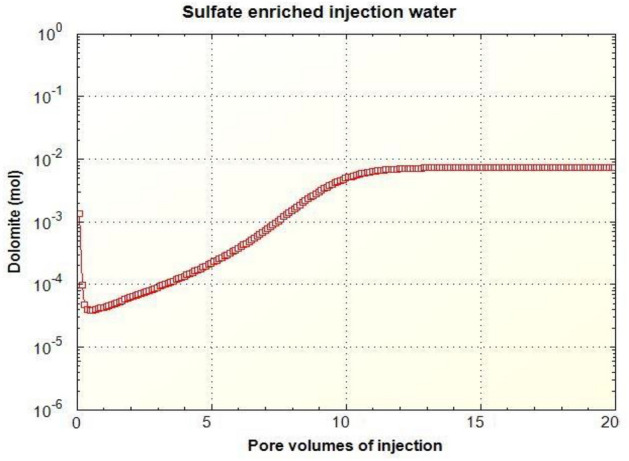
The corresponding plot of dolomite concentration profile in case of SW/40-4S injection water sample.

As can be seen in Figs. 11 and 13, as the dilution of water increases and the salinity of injected water decreases, the amount of carbonate rock site [CO3] increases. Also, the magnesium surface site [Mg]OH2 + in (SW-4S) candidate water sample and its diluted one (SW/40-4S) is lower than other water samples in Table 2. The reduction rate of magnesium surface site is faster in the diluted sample (SW/40-4S) than the (SW-4S) one. This trend shows the process of dolomitization, which can be seen in diluted and water samples with four times the sulfate (Fig. 14). As shown, in early stages of sulfate enriched injection (Sulfate Spike), dolomite concertation decreased due to slow rate of dolomitization precipitation reaction (reaction No. 7 in Table 7) compared to fast rate of decrease in [Mg]OH_2+_ surface site concentration. But whenever the injection time increased (more than 0.3 P.V.), rate of dolomitization overtake the rate of decrease in [Mg]OH_2+_ surface site concentration. Therefore, dolomite concentration increased and finally approach to stabilized value more than initial dolomite concentration of rock sample. This behavior is in line by other researchers’ findings such as Khurshid et al. stating that the adsorption of magnesium increases with the injection of sulfate-spiked water and consequently Ca2+ is replaced by Mg2+ in the rock^36^.

**Figure 15:**
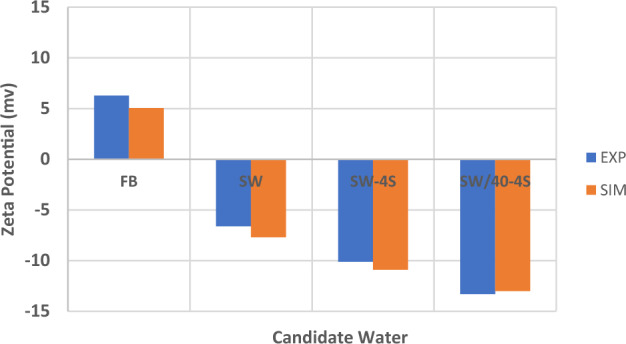
Comparison between calculated and experimental results of zeta potential in different candidate water and salinity levels of injection.

Figure 15 shows the summary of zeta potential calculated by the DLM model and compared with experimental measurements.

As shown in Fig. 15, the range of error is between 2 to 20% which is an improvement compared to other applied models in previous researches such as CD-MUSIC model in Takeya et al. (2019)^37^. The values of zeta potential are predicted based on the conventional models of electrical charge distribution (DLM, CD-MUSIC and BSM) in case of different candidate water (Table 9). It's clear that the error of prediction using DLM model is significantly less than the other models.

**Table 9:** Comparison of calculated values of zeta potential in different charge distribution models and in different candidate water and salinity levels of injection.

Electrical charge distribution model and EXP		FW	SW	SW-4S	SW/40-4S
Rock/brine	DLM	5.05	−7.7	−10	−13.2
	CD-MUSIC	22	−23	−28	−31
	BSM	19	−18	−25	−27
	Exp	6.28	−6.6	−10.1	−13.3
Oil/Brine	Exp	−37	−59	−57	−70

**Figure 16:**
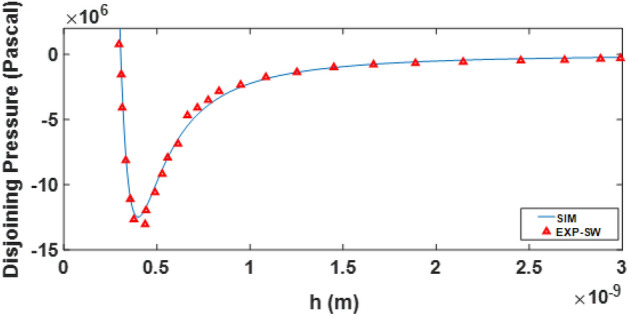
Validation of two-phase DLVO calculations based on a comparison of the results of disjoining pressure in the model with the results of Alshakhs in 2016.

DLVO theory calculation is done based on the calculated zeta potential of the PHREEQC model and using the Eqs. 9–14 to obtain the disjoining pressure curve and calculation of the contact angle (Eq. 15). In this study, unlike previous studies, the calculated zeta potential of the PHREEQC model is used to calculate the contact angle by the DLVO theory. Therefore, first, the calculations of the DLVO theory presented (Eqs. 9–14) were validated based on reproducing the disjoining pressure results of Alshakh’s study in 2016 (Fig. 16). It should be noted that the zeta potential data of the disjoining pressure input in Alshakh’s study is laboratory and non-computational.

**Figure 17:**
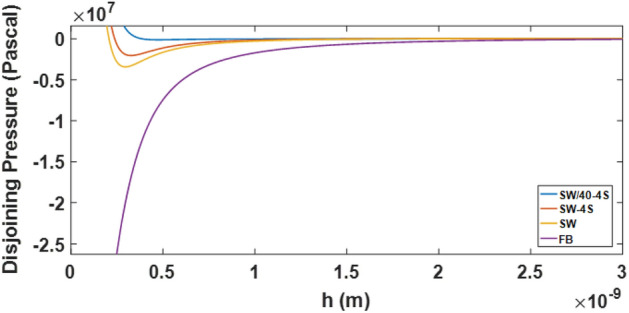
Prediction of disjoining pressure in different salinities based on DLVO theory.

In the following, based on the coupling of the developed geochemical model and valid DLVO theoretical calculations for the carbonate rock sample, the effect of salinity changes on the contact angle was investigated. Figure 17 shows the disjoining pressure curve at different injected salinities of candidate waters in Table 2.

**Figure 18:**
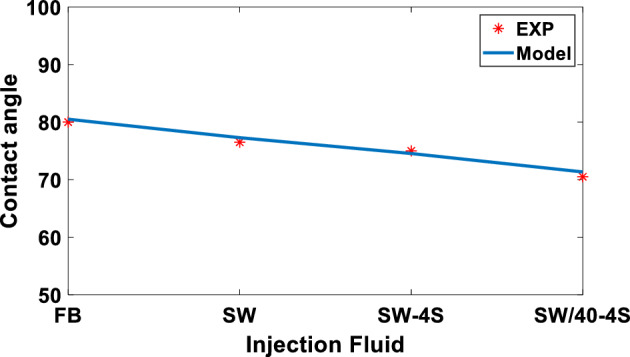
Comparison of contact angle changes based on DLVO theory modeling and static contact angle laboratory data at different salinities.

As can be seen in Fig. 17, if the salinity is reduced and the sample is diluted, the disjoining pressure increases and as a result the rock becomes more water wet. This trend of wettability changes is evident in Fig. 20. According to Fig. 18, a very good match is observed for the contact angle calculated based on DLVO theory modeling and the static contact angle experimental data at different salinities. The tabular results of Fig. 18 are shown in Table 10 in which the amount of contact angle has decreased in the range of 80 to 70 degrees with the decrease of salinity and increase of sulfate ion.

**Table 10:** Comparison of calculated values of contact angle and experimental measurements in different level of injection salinities.

Level of salinity/Results	Model	EXP
FW	80.5	80
SW	77.29	76.5
SW-4S	74.54	75
SW/40-4S	71.34	70.5

History match of Yousef et al.’s (2012) experimental data (test No. 1, Ref No. 3) is done to verify the dynamic performance of the developed static geochemical model (DLM). Rock and fluid data are imported in the two-phase transport in house code and coupled with the developed static geochemical model and DLVO calculations to generate the reactive flow modeling calculations in each time step. Three runs are done to compare our developed geochemical DLM model (sim C) with previous models (firstly with the geochemical model ignoring the charge distribution model (sim A) and secondly, with commonly used CD-MUSIC charge distribution model (Sim B)).

- Sim A does not use the surface sorption reactions (charge distribution models) and, consequently DLVO theory in each time step. It only uses the dissolution/precipitation reactions to modify the pore structure properties such as porosity.
- Sim B applies the CD-MUSIC charge distribution model in static geochemical model. This run considers the surface complexation reactions and is coupled with the DLVO calculation in each time step to estimate the contact angle. The contact angle is used as a geochemical interpolator to update the non-wetting phase relative permeability curve.
- Sim C is the same as sim B just only considering the DLM as the charge distribution model of static geochemical package.

**Figure 19:**
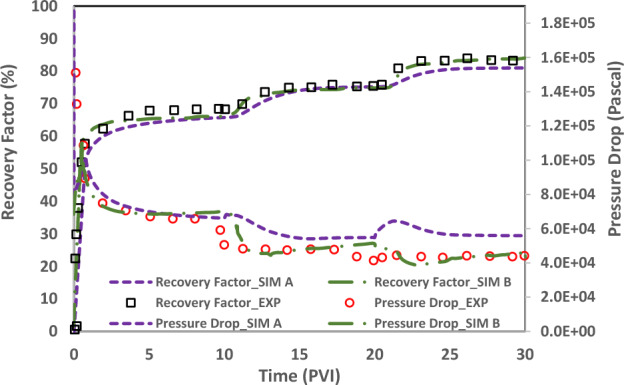
Comparison of oil recovery and pressure drop curves among two runs (SIM A and B) of the dynamic two-phase coupled geochemical and transport models.

**Figure 20:**
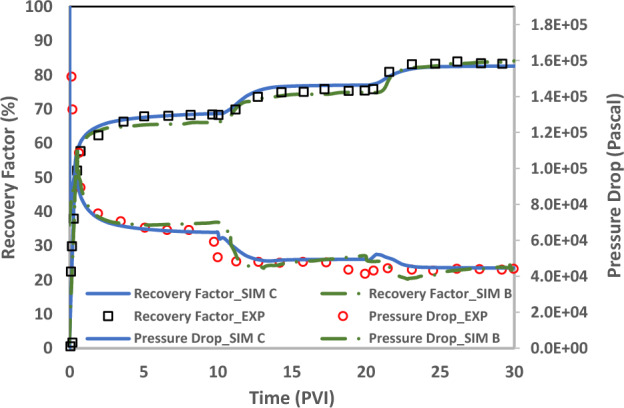
Comparison of oil recovery and pressure drop curves among two runs (SIM B and C) of the dynamic two-phase coupled geochemical and transport models.

Figure 19 shows the comparison of Sim A and Sim B indicating the significance of considering the surface sorption reactions (charge distribution models) especially in match quality of pressure drop curve. Figure 20 shows the comparison of Sim B and Sim C indicating the improvement in match quality of of both the recovery factor and pressure drop curves by application of more accurate charge distribution model (DLM).

Table 11 shows the standard deviation of simulation results from the experimental data by Eq. (17).

**Table 11:** Standard deviation (Sigma value- Eq. 16) of three runs (A, B and, C) from experimental data in the dynamic two-phase coupled geochemical and transport models.

Case	Sigma
Recovery Factor_SIM A	3236
Recovery Factor_SIM B	2692
Recovery Factor_SIM C	331
Pressure Drop_SIM A	1148
Pressure Drop_SIM B	468
Pressure Drop_SIM C	407

17 $$\mathrm{log}\,(Sigma)=\sqrt{\frac{\sum_{i=1}^{i=n}{\left({x}_{calculated}-{x}_{experiment}\right)}^{2}}{n-1}}$$

As shown in Fig. 20 and Table 11 in comparison of sim B and C, our developed model (sim C) can improve the match of the recovery factor with one order of magnitude (Sigma from 2692 to 331). In case of pressure drop curves, sim C enhance the match up to 15% relatively.

## Conclusion

Based on the zeta potential tests, accurate PHREEQC geochemical model is validated. Following results have been obtained based on coupling the DLVO theory and geochemical model for contact angle calculations in the carbonate rocks with low content of dolomite. The results are valid for similar brines in range of dilution (40 times) and sulfate enrichment (4 times) at 90 degrees Celsius:

- Diffused double layer (DLM) model has been used to determine the electrical charge distribution of rock/water and water/oil interfaces, in which the tuning parameters of surface sites are presented to match the experimental data. These parameters mentioned in Tables 6, 7, 8 can be used for rocks under the specified conditions of the paper without huge number of experiments.
- The use of the DLM charge distribution model will result in a more accurate calculation of the zeta potential than other models in the carbonate rock samples with low content of dolomite. The error data of the zeta potential modeled by DLM compared to the laboratory data is in the range of 2 to 20%. Results show that the error of prediction using DLM model is significantly less than the other models.
- Based on the application of the developed geochemical model and the DLVO theory, the contact angle value decreased in the range of 80 to 70 degrees with the decrease of salinity and increase of sulfate ion which is in acceptable agreement with the laboratory values.
- Based on the results of geochemical model, whenever the injection time increased (more than 0.3 P.V.), rate of dolomitization overtake the rate of decrease in [Mg]OH2 + surface site concentration. Therefore, dolomite concentration increased and finally approach to stabilized value more than initial dolomite concentration of rock sample.

## Supplementary Information

Supplementary Information.

## Data Availability

The data will be available upon request to Mohammad Parvazdavani as the first author (mohammadparvazdavani@gmail.com).
