# Supplementary material for: Prediction of electrostatic properties of reservoir rock in low salinity water injection into carbonate reservoirs
Source: Sci Rep. 2023 May 31;13:8782. doi: 10.1038/s41598-023-36032-4 (PMC10232532; doi:10.1038/s41598-023-36032-4)
Supplement: Supplementary file 1 — Supplementary Information. [file 41598_2023_36032_MOESM1_ESM.docx]

- Sensitivity analysis and optimization on change of contact angle versus salinity is done. The results are shown in figure SI.1 to obtain the optimum dilution factor of seawater sample. As shown in SI.1, 40 times dilution have been selected as the optimum dilution factor.

**SI.1. Change of contact angle versus salinity**

- The calculation of required salts in 1 and 2 Liter of solution for SW/40 (4S) brine recipe is presented in table SI.2. These amounts of salts lead to the stable ion composition. The same calculation procedure is done for other candidate injection brines.

SI.2. Required salts for SW/40 (4S) brine recipe

|  | **Salt, gr in 1 Liter Solution** | **Salt, gr in 2 Liter Solution** | ions | ion from salt, p.p.m |
| --- | --- | --- | --- | --- |
| **SrCl2.6H2O** | 0.0000 | 0.0000 | Sr | 0 |
| **MgCl2.6H2O** | 0.3429 | 0.6859 | Mg | 41 |
| **NaHCO3** | 0.0000 | 0.0000 | HCO3 | 0 |
| **CaCl2.2H2O** | 0.0459 | 0.0918 | Ca | 12.5 |
| **NaCl** | 0.7599 | 1.5197 | Na | 538 |
| **Na2SO4** | 1.4786 | 2.9573 | Cl | 602.7 |
| **LiCl** | 0.0000 | 0.0000 | SO4 | 1000 |
